# Multiple Chitin- or Avirulent Strain-Triggered Immunity Induces Microbiome Reassembly in Rice

**DOI:** 10.3390/microorganisms12071323

**Published:** 2024-06-28

**Authors:** Sauban Musa Jibril, Chun Wang, Chao Yang, Hao Qu, Xinyun Yang, Kexin Yang, Chengyun Li, Yi Wang

**Affiliations:** 1 State Key Laboratory for Conservation and Utilization of Bio-Resources in Yunnan, Yunnan Agricultural University, Kunming 650201, Chinayangchao78833@163.com (C.Y.); quhq1q@163.com (H.Q.);; 2 Yunnan-CABI Joint Laboratory for Integrated Prevention and Control of Transboundary Pests, Yunnan Agricultural University, Kunming 650201, China

**Keywords:** chitin, *Magnaporthe oryzae*, avirulent strain, bacterial community, innate immunity

## Abstract

*Magnaporthe oryzae* is one of the most important fungal pathogens of rice. Chitin and avirulent strains can induce two layers of immunity response, pathogen-associated molecular pattern (PAMP)-triggered immunity (PTI) and effector-triggered immunity (ETI), in rice with cognate *R* genes. However, little is known about the assembly of the rice microbiome induced by PTI and ETI in rice. In this study, we investigate the impact of continuous treatment of the avirulent *M. oryzae* strain with *AvrPi9* and chitin on the bacterial endophytic community of rice varieties harboring resistant gene *Pi9* and their antagonistic activity against rice blast fungus. Analysis of the 16S rRNA showed a significant increase in the diversity and microbial co-occurrence network complexity and the number of beneficial taxa—*Bacillus*, *Pseudomonas*, *Microbacterium*, and *Stenotrophomonas* spp.—following the chitin and avirulent strain treatments. The antifungal assay with bacterial endophytes recovered from the leaves showed few bacteria with antagonistic potential in rice treated with avirulent strains, suggesting that the sequential treatment of the avirulent strain decreased the antagonistic bacteria against *M. oryzae*. Moreover, we identified *Bacillus safensis* Ch_66 and *Bacillus altitudinis* Nc_68 with overall antagonistic activities in vivo and in vitro. Our findings provide a novel insight into rice microbiome assembly in response to different innate immunity reactions.

## 1. Introduction

Rice is a major and important staple food crop of the world, most of the world’s rice is produced and consumed in Asia, and China is the largest producer in the world. Rice blast, caused by a fungal pathogen, *M. oryzae*, is the greatest threat to rice production and causes a significant reduction in yield globally [1]. Thus, managing this devastating pathogen is paramount to global food security. Plants cope with pathogens’ attacks through innate immune systems composed of complex receptors that perceive the microbial pathogen attack and activate the immune response [2,3]. PAMP-triggered immunity (PTI) is the primary immune system that detects conserved microbial patterns (pathogen-associated molecular patterns, PAMPs) and triggers immunity via cell surface-localized pattern-recognition receptors (PRRs), such as receptor kinases (RKs) and receptor-like proteins (RLPs) [4,5,6]. Flagellin epitope flg22 and chitin are crucial components of bacterial and fungal PAMPs that are recognized by PTI receptors, such as FLS2 and LysM receptor kinase for immune response [7,8,9]. However, pathogens can successfully override PTI and evade the plant cell by secreting effector proteins [3,10]. The effector proteins are detected by intracellular nucleotide-binding leucine-rich repeat receptors (NLRs) and activate effector-triggered immunity (EFTI) [11]. Recognition of the pathogens’ effectors by NLRs results in a hypersensitive response and restricts pathogens to the site of infection [12]. PTI recognizes conserved PAMPs at the plant cell surfaces, and ETI recognizes the pathogens’ effector proteins to activate a stronger defense response. Recently, a plethora of evidence demonstrated that major components were shared in both PTI and ETI; PTI and ETI reinforce each other to induce more robust plant defense responses [13].

Plants are constantly associated with diverse beneficial and pathogenic microbial communities. The beneficial microorganisms form cooperative interactions with their host, and many of them play a role in plant growth and health [14]. The endophytic microbiome colonizes the plant’s intercellular spaces and possesses incredible growth promotion and pathogen suppression. The endophytic microbiome can offer further protection against pathogens by enhancing members’ repertoire of genetic machinery to produce enzymes and secondary metabolites against the pathogen, and by modulating host genes involved in the defense against biotic stresses [15,16]. In addition, the beneficial microbes are exposed to the plant immune system and have to modulate the innate immune system to colonize their host [17]. To colonize their host, symbiotic microbes evade PRR recognition through modification of PAMP and secretion by the effector protein, such as MiSSP7 by *Laccaria bicolor* and NGR234 by *Rhizobia* spp. [18,19,20]. Fungi prevent PTI activation by conversion of chitin to chitosan; this mechanism is also employed by beneficial fungi for plant colonization [21,22].

Beneficial bacterial communities could have an impact on plant immunity through the regulation of immune receptor activity, transcription factors, the activation of mitogen-activated protein kinase cascades to enhance disease resistance, and reactive oxygen species signaling [21]. In this regard, a previous study showed that *Arabidopsis thaliana* leaves respond differently to colonization of commensal bacterial *Sphingomonas melonis* Fr1 (S.Fr1) and *Methylobacterium extorquens* PA1 (M.PA1). While the former affected the expression of numerous genes, the latter activated the genes related to defense responses that are partly elicited by *Pseudomonas syringae* DC3000 [23]. However, the role of the plant immune system in shaping the microbial community is not fully understood, especially the characters of microbiome reassembly induced by PTI or ETI in plants. In this study, we investigated the effects of sequential treatment of chitin and avirulent strains on the rice bacterial endophytic community encoding resistance gene, *Pi9.* We further isolated bacterial strains and carried out an antifungal assay against the rice blast pathogen in both in vitro and in vivo conditions.

## 2. Materials and Methods

### 2.1. Inoculation with M. oryzae or Chitin

Pot experiments were conducted to assess the effects of chitin or avirulent *M. oryzae* on the rice bacterial endophytes. The IRBL9-W rice seeds were planted in the pots, which were filled with sterilized soil using a randomized complete block design, and 21-day-old seedlings were used for inoculation. Chitin (80 nM) or avirulent *M. oryzae* (1 × 10^5^ spores/mL) was sprayed on different rice leaves, respectively. The inoculation was performed three times 7 days after inoculation. For the first and second inoculations, samples were collected 3 and 7 days post-inoculation, followed by the next inoculation and subsequent sampling. Rice seedlings foliar-sprayed with sterilized distilled water (ddH_2_O) were used as the control (CK). The control samples were collected at the beginning and end of the sequential inoculations of chitin or avirulent strains. Ch indicates the rice seedlings treated with chitin and Mo indicates the avirulent *M. oryzae* strain treatment, whereas NC and NE are the initial and final control, respectively. Three replicates were used for each sample (Figure S1).

### 2.2. Genomic DNA Extraction, 16S rRNA Sequencing and Analysis

Total genomic DNA was extracted from the 36 chitin- and avirulent strain-treated leaf samples and 6 initial and final controls with the PowerSoil DNA Isolation Kit (DNeasy PowerSoil Kit, QIAGEN, Hamburg, Germany) following the manufacturer’s instructions. The primers 799F (5′-AACMGGATTAGATACCCKG-3′) and 1193R (5′-ACGTCATCCCCACCTTCC-3′) were used to amplify the target 16S rRNA V5-V7 fragment by PCR. The purified fragments were sequenced on an Illumina PE300 platform (Illumina, San Diego, CA, USA), according to the standard protocols by Majorbio Bio-Pharm Technology Co. Ltd. (Shanghai, China). The sequence reads were processed in Qiime2 [24]. The high-quality sequences were clustered into amplicon sequence variants (ASVs) using the DADA2 plugin in the Qiime2 pipeline based on recommended parameters [24]. Naive Bayes was used to determine the ASVs’ taxonomy against the SILVA database [25].

### 2.3. Bioinformatics Analysis

The phyloseq package was used to calculate the alpha diversity indices (Shannon and observed species). The results of the alpha diversity were visualized using the ggplot2 R package [26]. Significant differences between the indices were calculated using IBM SPSS 20.0 (SPSS Inc., Chicago, IL, USA) according to a Duncan multiple test (*p* < 0.05). Beta diversity was determined using Nonmetric multidimensional scaling (NMDS) based on weighted UniFrac distance matrices. The dissimilarities among the communities were determined using Permutational multivariate analysis of variance (PERMANOVA) using Adonis of the vegan package [27]. Chord diagrams were used to visualize the relative abundance of genera using Circos [28]. A Kruskal–Wallis test was used to calculate the significant difference among the bacterial genera (*p* < 0.05). A microbial co-occurrence network analysis was conducted using the Sparse Correlations for Compositional data algorithm (SparCC) package at the ASV level (*p* > 0.05 and correlation coefficient > 0.3). The network was visualized in Gephi (v0.9.2) [29]. Network nodes with a high degree of closeness centrality values were assigned as hub nodes [30]. 

### 2.4. Isolation and Identification of Endophytes

The bacterial strains were isolated from rice leaves among CH, Mo, NC, and NE samples. Briefly, the leaf samples from each treatment were surface-sterilized by soaking in 70% alcohol (*v*/*v*) for 30 s, followed by 0.1% mercuric chloride solution for 8 min, and finally rinsed with sterile distilled water 8 times. The sterilized leaves were grounded in a sterilized mortar and pestle, placed into a 50 mL centrifugal tube with 10 mL MgCl_2_ (10 mM), and placed into thermostatic oscillator culture (28 °C, 120 rpm) for 24 h and then 10× serial diluted using sterile aqueous solution 10, 100, 1000, and 10,000 times. The serially diluted samples were placed into the TSB medium by the coating plate method and incubated (25 °C) for 3–4 days. The individual bacterial colonies were aseptically subcultured for further purification and 36 pure cultures were obtained. The purified bacterial cultures were stored in 30% glycerol solution at −80 °C. To obtain the accurate classification of the rice leaves’ endogenous isolates, 16s genomic DNA was amplified and sequenced. The sequences were BLAST searched on the National Center for Biotechnology Information (NCBI) database and we obtained the closest species.

### 2.5. In Vitro Assays to Evaluate the Antifungal Activities of the Bacterial Strains

To evaluate the antifungal activities of the isolated strains, four pathogenic fungi, *M. oryzae*, *Botrytis cinerea*, *Rhizoctonia solani*, and *Bipolaris maydis,* were used in a plate confrontation method on a CM plate [31]. About 7 mm fungal plugs of the pathogens were placed at the center of the CM plate and each bacterial strain was inoculated about 2 cm from the center at four sides of the plate incubated at 28 °C. The antifungal potential was assessed by measuring the inhibition zones of the strains at 7 days post-inoculation (dpi). The experiment was repeated four times with three replicates in each treatment.

### 2.6. Greenhouse Assays

We then tested the antifungal activity of the 4 *Bacillus* strains that showed strong antifungal effects on 21-day-old rice seedlings by single and mixed inoculations. For the single inoculation, a spore suspension (1 × 10^5^ spores/mL) of individual bacterial strains was inoculated on susceptible rice seedlings (Lijiangxintuanheigu, LTH) and followed by inoculation of *M. oryzae* one day later. For the mixed inoculation, the spore suspension of each strain was mixed with 10 mL of rice blast spore suspension and inoculated individually with LTH by evenly spraying with the mixed spore suspensions, while the controls (CKs) were foliar-sprayed with spore suspensions. The inoculated seedlings were kept in dark conditions under high humidity and transferred to the growth chamber after 24 h. After 7 days of inoculations, the disease incidence was assessed using a 0–5 scale [32].

The disease index was calculated using the formula

$$\frac{\sum (rating\;scale\times number\;of\;diseased\;leaves)}{Total\;number\;of\;plants\times 5}\times 100$$

## 3. Results 

### 3.1. Effects of Chitin or Avirulent M. oryzae Treatments on Diversity Structure of Rice Bacterial Endophytic Community

**Figure 1:**
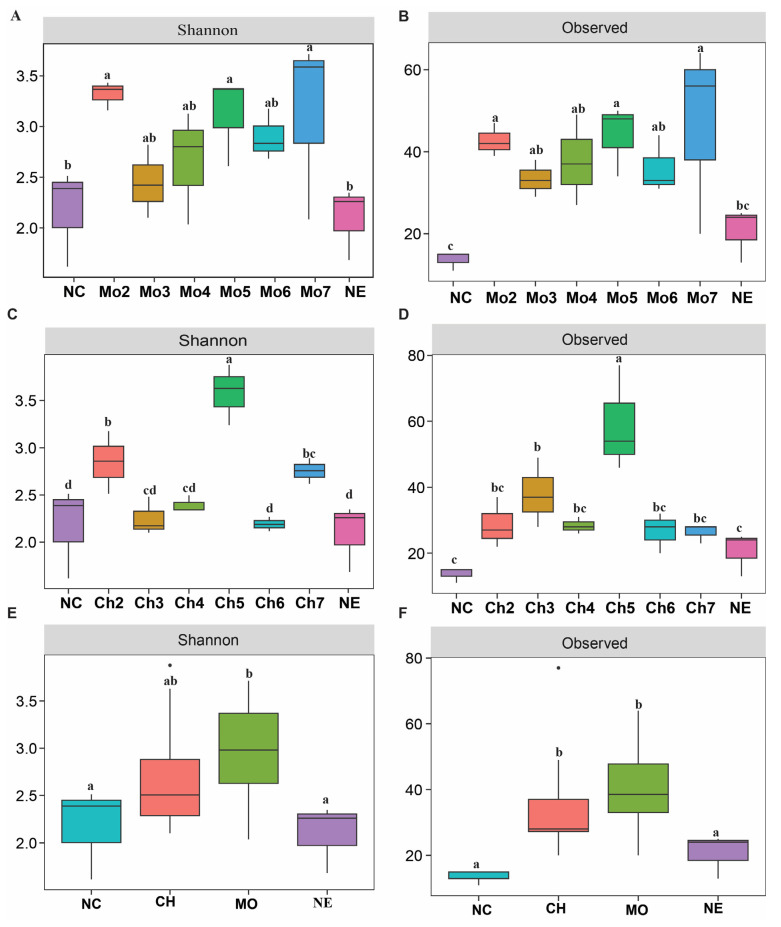
Alpha diversity indices (Shannon and observed species) of the rice bacterial community under sequential treatment of avirulent *M. oryzae* (**A**,**B**) and chitin (**C**,**D**). The box plots showing the changes in the alpha diversities in avirulent *M. oryzae* and chitin treatments analyzed at group level (**E**,**F**). NC and NE are the initial and final control, respectively. Different lowercase letters represent the significant differences among treatments (Duncan multiple test range, *p* < 0.05).

A total of 253 ASVs were obtained after the removal of ASVs assigned to mitochondria and chloroplasts. The differences in alpha diversity indices of the bacterial communities were assessed by the Shannon index and observed species. For the avirulent strain treatment, the Shannon index and observed species were significantly higher in the treatment compared with the control (NC and NE). In general, the indices increased significantly in Mo2 with a decrease in Mo3 and a gradual increase in Mo4, Mo5, and Mo7, with a slight decrease in Mo6 (Figure 1A,B). For the chitin treatment, there was a significant increase in observed species in Ch2, Ch3, and Ch5 and a gradual decrease in Ch4, Ch6, and Ch7, respectively, whereas no significant differences were observed between NC and NE (Figure 1C,D). Compared with control, chitin or avirulent strain treatment exhibited a significant higher observed species index (Figure 1E,F).

**Figure 2:**
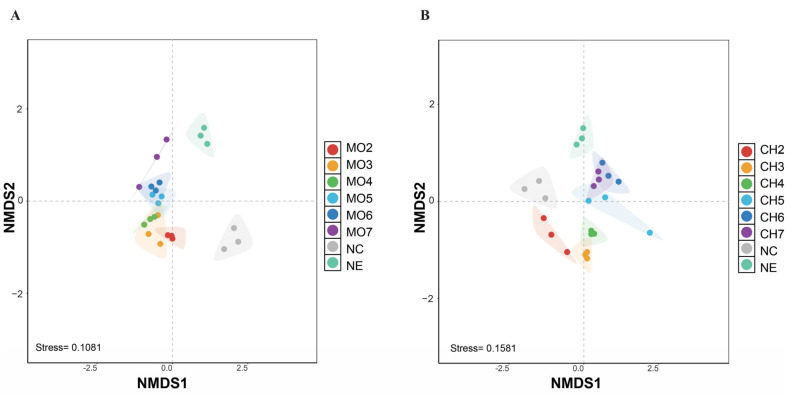
Bacterial beta diversity in the rice analyzed using Nonmetric multidimensional scaling (NMDS) ordination of bacterial communities. NMDS presenting the pattern of bacterial communities under avirulent strain of *M. oryzae* (**A**) and chitin treatments (**B**).

Nonmetric multidimensional scaling (NMDS) based on the Bray–Curtis dissimilarity matrix revealed the variations in the bacterial communities between avirulent *M. oryzae*, chitin sequential treatments, and the initial and final control, indicating that the two treatments induced the changes in the diversity of the bacterial communities (Figure 2A,B). Permutational multivariate analysis of variance (PERMANOVA) indicated that the endophytic communities differed under different treatments (chitin, R^2^ = 0.6773, *p* < 0.001 and *M. oryzae* avirulent strain, R^2^ = 0.7058, *p* < 0.01) (Tables S1 and S2). Comparing the chitin and avirulent treatment groups, NMDS analysis shows a clear separation of bacterial communities between the two treatments and the NC and NE (Figure S2). This suggested that the diversity of the rice bacterial community was influenced by the two treatments.

### 3.2. Changes in the Relative Abundance of Endophytic Bacteria under Chitin or M. oryzae Avirulent Strain Treatments

**Figure 3:**
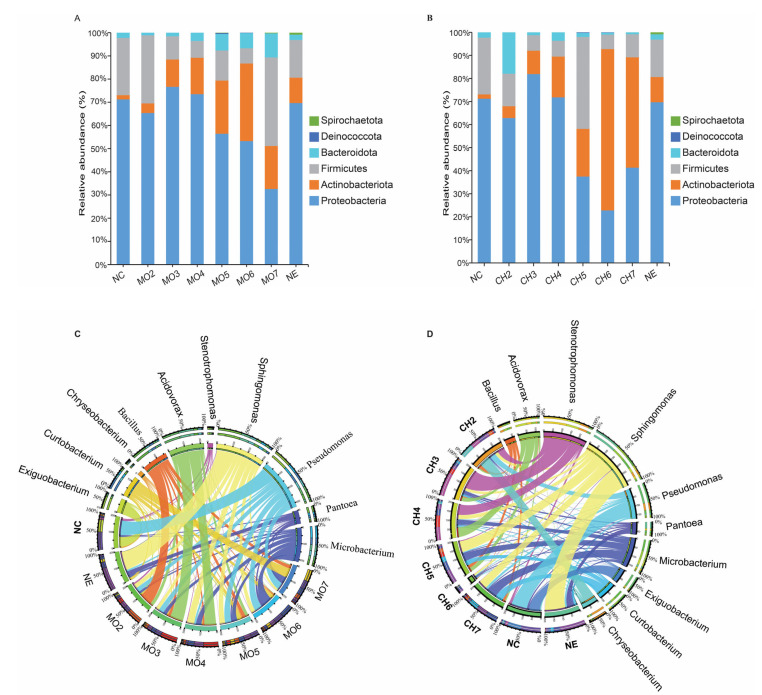
The taxonomic composition of rice-associated bacterial communities in chitin and avirulent *M. oryzae* treatments. Distribution of the top-10 most abundant phyla in avirulent strain (**A**) and chitin treatment (**B**). Chord diagrams showing the changes in the relative abundance of the top-10 genera in avirulent *M. oryzae* (**C**) and chitin treatments (**D**).

The relative abundance of the bacterial community at the phylum and genus levels was analyzed across the two treatments. In both the chitin and avirulent strain treatments, the most abundant bacterial phyla were Proteobacteria, Actinobacteria, Firmicutes, and Bacteroidota. In the *M. oryzae* avirulent strain treatment, the relative abundance of Proteobacteria increased at the first and second stages and gradually decreased at the later stages. Bacteroidota increased slightly with the treatment, whereas the phyla Actinobacteriota and Firmicutes had no consistent patterns across the treatments (Figure 3A). For the chitin treatment, the responses of the bacterial phyla were not consistent patterns across the treatment group (Figure 3B). For both the initial and final controls, the phyla Proteobacteria, Actinobacteriota, and Firmicutes dominated the composition with total average abundances of 85–89%.

A chord diagram was used to visualize the relative abundance of bacterial communities at the genus level. The most abundant genera in the initial control (NC) are *Pseudomonas* and *Exiguobacterium*. Final control (NE) was dominated by genera *Sphingomonas* and *Pantoea*. The response of rice endophytes at the genus level after the two treatments showed inconsistent patterns across the six inoculation stages. In the *M. oryzae* avirulent strain treatment, the relative abundance of *Sphingomonas* increased gradually and reached a peak in Mo4; the genus *Bacillus* was the most abundant at Mo2, while *Acidovorax* was relatively abundant in Mo3 and Mo4. In Mo7, *Curtobacterium*, *Bacillus*, *Microbacterium*, and *Pseudomonas* were the most dominant genera (Figure 3C). In the chitin treatment, the relative abundance of *Microbacterium* increased gradually and reached a peak at the Ch7 stage. The abundance of *Stenotrophomonas* increased in Ch2 to Ch4 and reached a peak in the Ch3 stage. The relative abundance of *Acidovorax* initially increased and was highest at Ch4, but gradually decreased at later stages (Figure 3D). 

To precisely assess the response of the bacterial community to the chitin and avirulent strain treatments, we compared the relative abundance of phyla and the top-ten genera at the group level by combining the treatment of each sample into one group. We observed an increase in the phylum Actinobacteriota and a decrease in Proteobacteria following the two treatments (Figure S3). A significant increase in the abundance of genus *Bacillus* was observed under chitin, and genus *Microbacterium* was induced by the avirulent strain treatment (Figure S4). Taken together, chitin and an avirulent strain of *M. oryzae* induce a shift in the abundance of beneficial bacterial endophytes.

### 3.3. Effects of Chitin or M. oryzae Avirulent Strain on Microbial Co-Occurrence Networks

**Figure 4:**
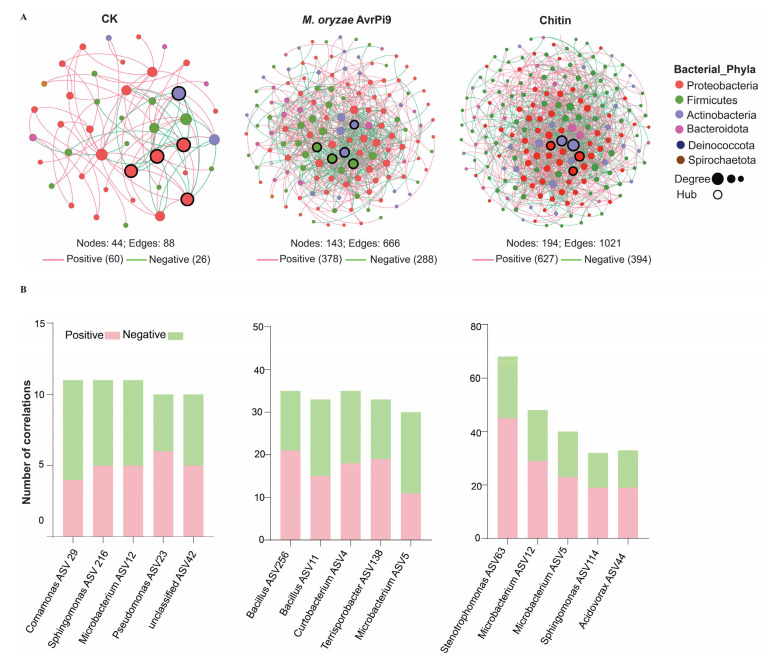
Co-occurrence network analysis of rice bacterial endophytes under different treatments. Visualized microbial association showing a higher network complexity in chitin and *M. oryzae* treatments than CK (**A**). The interaction of the top-5 hub taxa with other nodes showing the variation in positive and negative correlation (**B**). The nodes are colored according to taxonomic annotation at the phylum level. The size of the node shows the degree. Positive correlations are shown in pink color and negative in green color.

Due to the effects of the chitin or avirulent treatments observed on the microbial community composition, we constructed a microbial co-occurrence network at the ASV level for each treatment group. The network complexity assessed through topology characteristics indicated that the number of nodes, edges, and average degrees were significantly higher in the chitin and *M. oryzae* avirulent strain treatments compared to the control (Figure 4A, Table S3). Considerable differences between the microbial co-occurrence networks suggest a difference in responses of innate immunity of bacterial endophytes between chitin and avirulent strain treatments. We suggest that chitin detected by PTI led to a more complex network than the avirulent strain that could trigger ETI, resulting in weaker interaction and complexity. Furthermore, the interactions between the nodes were dominated by negative–positive links in the CK; in contrast, enhanced positive correlations were observed in the chitin and avirulent *M*. *oryzae* networks (Figure 4A). To evaluate the changes in the microbial interactions between the two treatments and the control, taxa with the most interactions with other nodes were identified. Hub taxa are known to have a significant impact on microbial interactions; therefore, we assigned the top-five hub nodes with a high degree of closeness centrality to hub taxa. A variation of hub taxa was found in the treatment group compared with the CK (Figure 4B). Beneficial bacterial genera such as *Bacillus* (ASV 256 and 11) and *Microbacterium* were assigned as hub taxa in the network of avirulent strain of *M. oryzae* treatment whereas *Microbacterium* (ASV5 and 12), *Sphingomonas* and *Stenotrophomonas* were assigned as hub taxa chitin treatments.

### 3.4. Isolation and Identification of Endophytic Strains

Thirty-six bacterial strains were isolated based on morphological and molecular analyses and the results were further confirmed by a BLAST search in NCBI. Based on the NCBI BLAST search, fifteen bacterial isolates were recovered from chitin treatment, while four, eleven, and six isolates were recovered from the avirulent treatments NC and NE, respectively. The isolates were classified into 8 genera, belonging to 13 strains of *Microbacterium*, 11 strains of Bacillus, 4 strains of *Rhodococcus*, and 3 strains of *Brevundimonas*. There were 2 strains of *Sphingomonas*, 1 strain of *Kocuria*, 1 strain of *Paenibacillus*, and 1 strain of *Deinococcus* (Table S4). 

### 3.5. Bacillus Strains Showed Strong Antifungal Activity against M. oryzae and Other Fungal Pathogens

**Figure 5:**
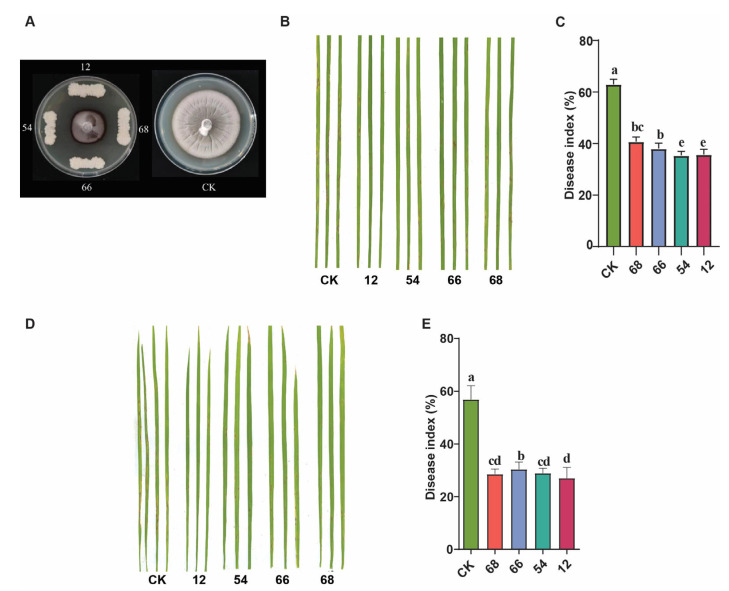
Antagonistic activity of four *Bacillus* strains against rice blast pathogen. Effect on mycelial growth (**A**). Effect on the pathogenicity by inoculation of *M. oryzae* combined with individual strain (mixed inoculation) (**B**); disease index (**C**). Effect on the pathogenicity by inoculation of bacterial strain and followed with *M. oryzae* one day later (**D**); disease index (**E**). The disease index was assessed at 7 dpi. Different lowercase letters indicate significant differences between treatments according to Duncan’s test *p* < 0.05.

To test the antagonistic activity of the bacterial strains, fungal pathogens *M. oryzae*, *Botrytis cinerea*, *Rhizoctonia solani*, and *Bipolaris maydis* were treated under different plates. Based on the rate of inhibition of mycelial growth in the CM plate, two strains of *B. safensis* (Ch_12 and Ch_66) from the chitin treatment, one strain from NE (NE_54), and one strain of *B. altitudinis* NC_68 from NC displayed strong antifungal potential against *M. oryzae* and some other pathogens (Figure S5). All four strains showed excellent antifungal activity against *M. oryzae* as indicated by mycelial inhibition compared to the control (Figure 5A). However, the bacterial strains isolated from the avirulent strain treatment samples did not show antifungal activity against *M. oryzae* but they showed antifungal activity for other pathogen (Figure S5C).

Furthermore, the LTH rice seedlings were inoculated by two methods. One is the spray of the bacterial strains for one day, followed by *M. oryzae* after one day. The other method is to inoculate the mixture suspension containing the bacterial strains with a spore suspension of *M. oryzae* simultaneously. The results showed a significant reduction in the number of lesions and the disease index (DI) in both methods compared with the CK (Figure 5B–D). Our results demonstrated that the rice bacterial endophytic strains could inhibit blast fungal growth under laboratory and greenhouse conditions. However, *B. safensis* Ch_66 showed an overall higher antifungal activity against the rice blast fungus; this is possibly due to the presence of specific antifungal compounds.

## 4. Discussion

Plants are constantly in contact with diverse groups of beneficial and pathogenic microbes; plants perceive and respond to pathogens by complex innate immune systems. Pattern recognition receptors present on the cell surface detect pathogen-associated molecular patterns and initiate the first layer of defense, pattern-triggered immunity [3,5]. Pathogens interfere with the PTI by injecting effector proteins and could activate effector-triggered immunity [11]. Previous studies have demonstrated that plant immunity and associated microbiota work simultaneously in defense against pathogen infections [21,23]. In the present study, we reported the response of the bacterial endophytic community to the chitin and *M. oryzae* avirulent strain treatments.

We utilized rice harboring the *Pi9* rice blast resistance gene and we found that the alpha diversity was significantly higher under the chitin and avirulent strain of *M. oryzae* compared to the controls. The microbial diversity indices were higher in the avirulent than in the chitin treatment (Figure 1E,F). We suggest that the increase in the microbial diversity in the avirulent treatment was due to the activation of both the PTI and the ETI, resulting in corresponding cross-talk between innate immunity and microbiota for the suppression of pathogenic particles. Previous studies have reported that chitin induces PTI defense responses characterized by the activation of the mitogen-activated protein kinase (MAPK) pathway, while the avirulence effector AvrPiz-t from the rice blast fungus *M. oryzae* was induced in both PTI and ETI [33,34,35,36]. Previous findings showed that infection by *Pseudomonas syringae* pv. tomato on an *Arabidopsis* quadruple mutant (mfec) defective in the PTI signaling components MIN7 and CAD1 brings about a decrease in the Shannon index and microbial diversity and causes chlorosis and necrosis on the leaf tissue compared with the non-mutant Col-0 plants [37]. These results further support our findings that plant innate immunity together with an associated microbiome are crucial for plant immunity.

Furthermore, the relative abundance of the bacterial community was greatly affected by the chitin and avirulence treatments; the relative abundance of *Pseudomonas*, *Bacillus*, *Sphingomonas*, *Microbacterium*, *Stenotrophomonas*, and *Acidovorax* significantly increased under the avirulence treatment. Numerous previous studies reported that members of the genera *Pseudomonas*, *Bacillus*, *Sphingomonas*, and *Microbacterium* are well known for their growth promotion potentials and antagonistic activities against plant pathogens [38,39,40,41]. Several studies suggest the importance of the increase in the abundance of a beneficial microbiome following biotic or abiotic stresses. A previous study demonstrated that a shift and increase in the abundance of beneficial bacterial endophytes *Pantoea*, *Pseudomonas*, and *Curtobacterium* following bacterial blight infection in rice was due to the ability of the plant microbiome to respond to pathogen infection [42]. Members of the genus *Sphingomonas* have been reported to induce protection against *P. syringae* by suppressing disease symptoms and lessening the pathogen’s growth [43]. It is reported that members of the genus *Stenotrophomonas* can engage in a beneficial interaction with plants and promote growth, particularly under stressful conditions [44,45,46]. Our results are in accordance with the previous findings that PTI signaling components CAD1 and MIN7 control the assembly, diversity, and composition of the *Arabidopsis* leaf bacterial endophytic community [47].

Analysis of the microbial co-occurrence network revealed that the chitin and avirulent treatments could increase microbial interactions and complexity and enhance positive interactions among the bacterial endophytes. It has been reported that network complexity and increases in positive interactions in response to pathogenic infections indicate mutualistic relationships and cooperation that can give rise to pathogen resistance [48,49]. A previous study showed that intense competition among microbial communities could result in plant development, nutrient acquisition, and the activation of immune signals [21]. Hub microorganisms can be used to optimize the function of the plant-associated microbiome and are essential in organizing the assembly and function of host–microbe interactions [50,51,52]. Beneficial taxa such as *Stenotrophomonas*, *Bacillus*, *Microbacterium*, and *Sphingomonas* were identified as hub taxa. *Stenotrophomonas*, *Bacillus*, and *Microbacterium* play an important role in stress tolerance and pathogen suppression through antibiosis and growth promotion [46,53,54]. However, we obtained the antagonistic bacteria with excellent antifungal activities from all samples, except for the seedlings treated with an avirulent strain of *M. oryzae* which isolated bacteria showed antifungal activities against pathogens except *M. oryzae*. Moreover, there might be some key immunity-related microbes in avirulent strain-treated samples that are difficult to isolate. Thus, there are largely unknown aspects to be revealed about the connections between plant immunity and the microbiome.

## 5. Conclusions

Overall, our study demonstrated that sequential foliar spraying of chitin or an avirulent strain of *M. oryzae* could trigger innate immunity that could lead to the increase in the bacterial community composition and overall microbial interactions. The two treatments enhanced the diversity of beneficial bacterial taxa that could play a crucial role in the suppression of the growth of pathogens. The antifungal assay of the native rice bacterial endophytes isolated after the treatments revealed several species of *Bacillus*, particularly *B. safensis* Ch_66, from chitin-treated seedlings that could significantly inhibit the mycelial growth and decrease the pathogenicity and the disease index of the rice blast fungus. Findings from this study could improve our knowledge of the interaction of plant innate immunity with the associated microbiome and the roles of native microbiota in disease management.

## Data Availability

The 16S rRNA sequencing data can be found in NCBI SRA under the BioProject number PRJNA1061119.

## Supplementary Materials

The following supporting information can be downloaded at https://www.mdpi.com/article/10.3390/microorganisms12071323/s1: Figure S1: The experimental route adopted for inoculation of chitin and *M. oryzae* and sample collection; Figure S2: Impact of chitin and avirulent *M. oryzae* treatments on the beta diversity; Figure S3: Comparison of the relative abundance of bacterial phylum at the group level; Figure S4: Comparison of genus relative abundance for group treatment; Figure S5: Effects of Bacterial strains on the fungal pathogens mycelial growth. Table S1: Results of PERMANOVA by Adonis of bacterial endophytes treated with chitin; Table S2: Results of PERMANOVA by Adonis of bacterial endophytes treated with *M. oryzae*; Table S3: Microbial co-occurrence network characteristics; Table S4: Sequence alignment of 16S rDNA genes in isolated and purified rice endophytic bacteria.
