# Supplementary material for: Multiple Chitin- or Avirulent Strain-Triggered Immunity Induces Microbiome Reassembly in Rice
Source: Microorganisms. 2024 Jun 28;12(7):1323. doi: 10.3390/microorganisms12071323 (PMC11279204; doi:10.3390/microorganisms12071323)
Supplement: Supplementary file 1 [file microorganisms-12-01323-s001.zip › microorganisms-3069141-supplementary.pdf]

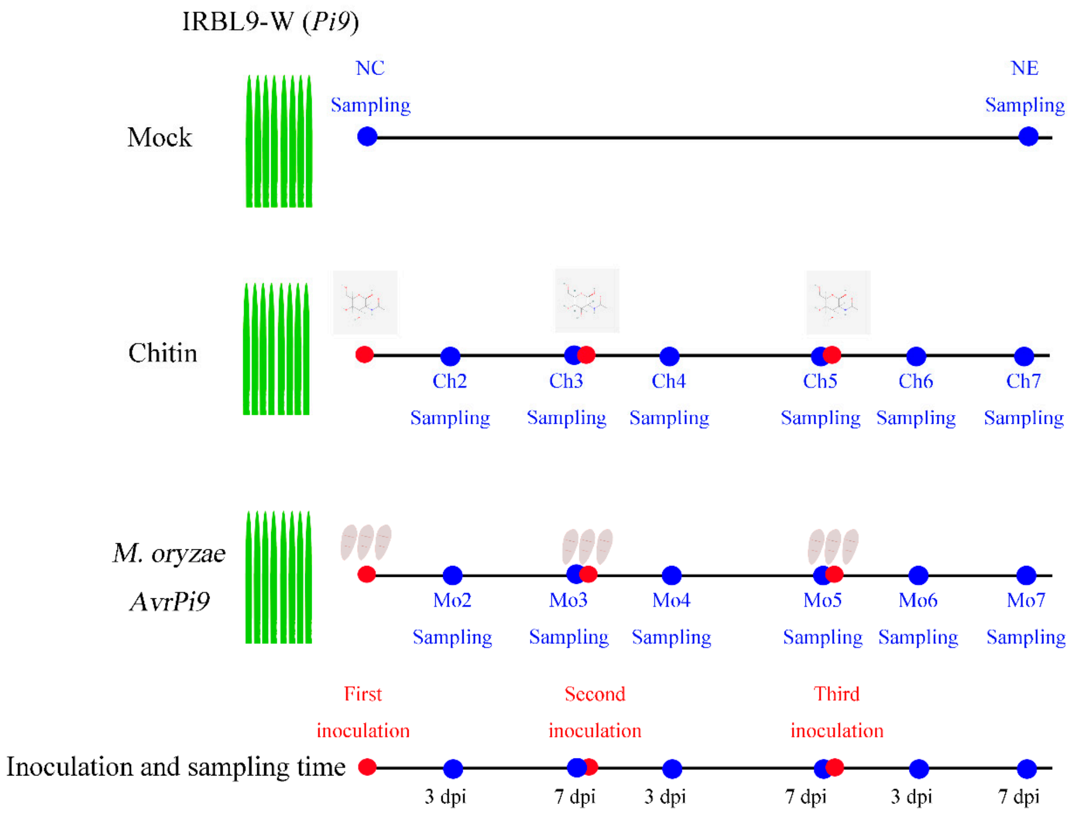

Figure S1. The experimental route adopted for inoculation of chitin and *M. oryzae* and sample collection. Water was used in the control (Mock).

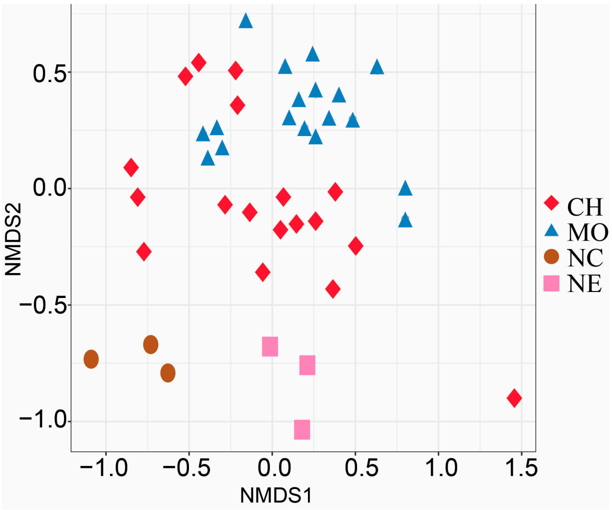

Figure S2. Impact of chitin and avirulent *M. oryzae* treatments on the beta diversity. Nonmetric multidimensional scaling (NMDS) ordination of bacterial communities based on Bray Curtis of bacterial community for group samples.

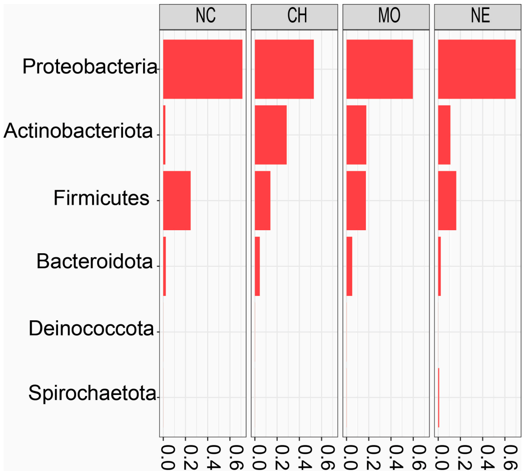

Figure S3. Comparison of the relative abundance of bacterial phylum at the group level.

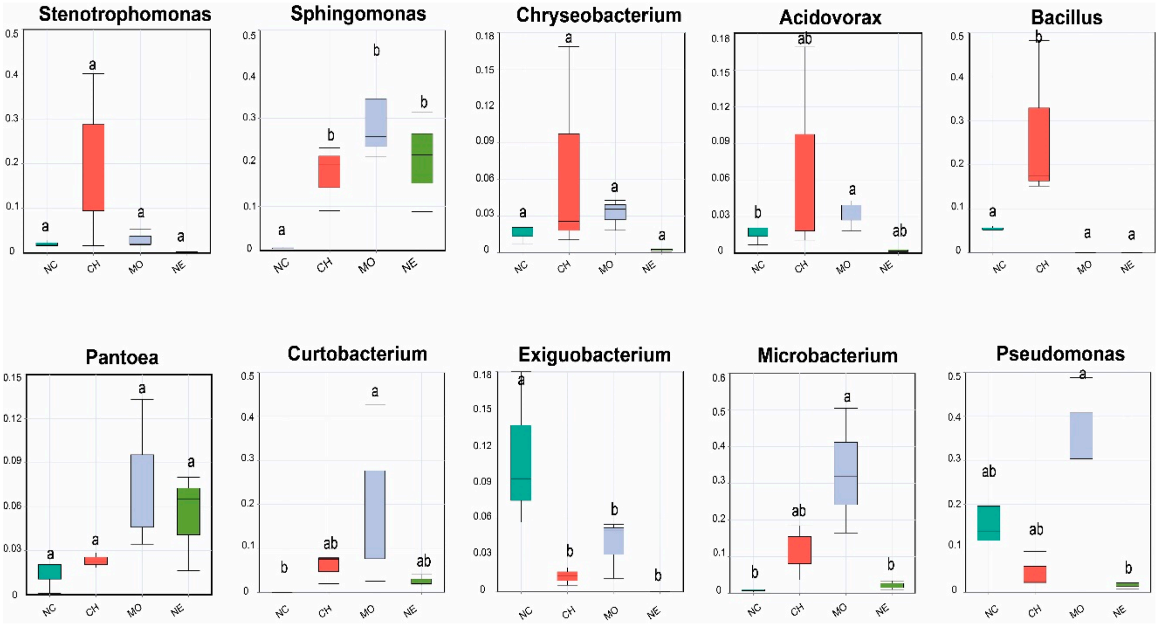

Figure S4. Comparison of genus relative abundance for group treatment. Different lowercase letters indicate the significant differences among the treatments (Duncan multiple test range, $p < 0.05$).

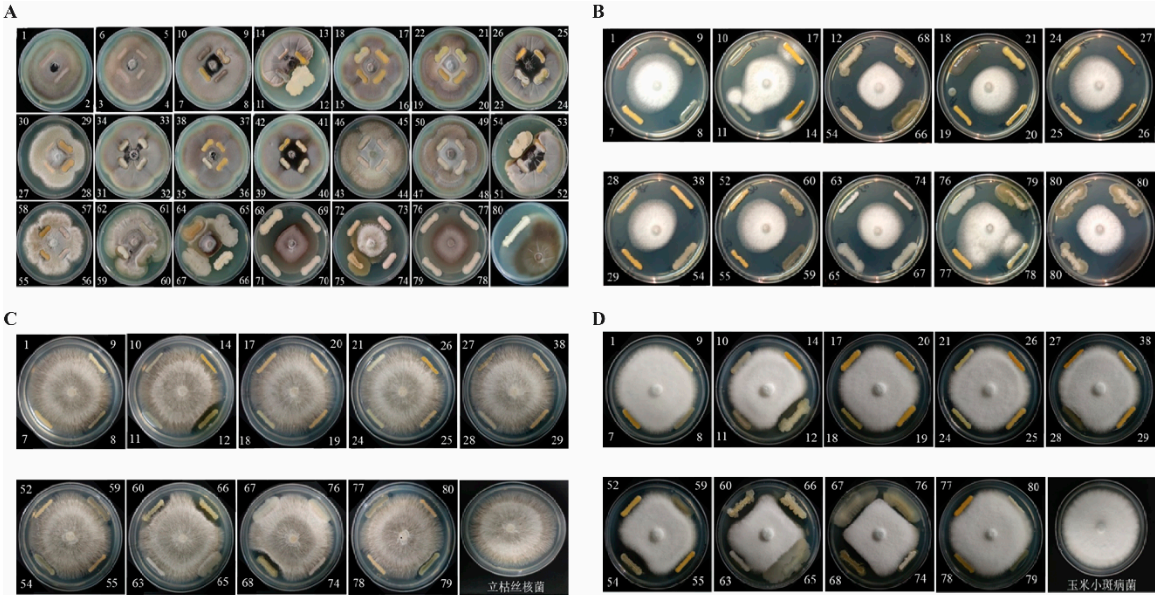

Figure S5. Effects of Bacterial strains on the fungal pathogens mycelial growth (A) *M. oryzae* (B) *Botrytis cinerea,* (C) *Rhizoctonia solani,* and (D) *Bipolaris maydis*

Table S1. Results of PERMANOVA by Adonis of bacterial endophytes treated with chitin.

	Df	**SumofSqs**	**R^2**	**F**	**Pr(>F)**	
Site	7	5.5249	0.67739	4.7993	0.001	***
Residual	16	2.6313	0.32261			
Total	23	8.1662	1.00000			

Table S2. Results of PERMANOVA by Adonis of bacterial endophytes treated with *M. oryzae*

	Df	**SumofSqs**	**R^2**	**F**	**Pr(>F)**	
Site	7	5.1925	0.70588	5.4857	0.001	***
Residual	16	2.1635	0.29412			
Total	23	7.3560	1.00000			

Table S3. Microbial co-occurrence network characteristics

Networks	No. Node	No. of edges	Positive edge	Negative edge	Average degree	Modularity	Avg. clustering coefficient	Avg. path length
CK	44	88	60	26	3.909	0.494	0.426	3.404
M.oryzae	143	666	378	288	9.315	0.355	0.351	2.849
Chitin	194	1021	627	394	10.526	0.304	0.224	2.763

Table S4. Sequence alignment of 16S rDNA genes in isolated and purified rice endophytic bacteria

Strain ID	Treatment	GenBank closest	Species	Query Cover	Similarity	Accession
1	Ch	*Deinococcus aquaticus* strain FR100	*Deinococcus aquaticus*	100%	100.00%	MH504183.1
7		*Microbacterium testaceum* strain DK33	*Microbacterium testaceum*	100%	99.92%	MT534540.1
8		*Brevundimonas* sp. strain SH-3.2-R-10	*Brevundimonas* sp.	100%	100.00%	MN784238.1
9		*Microbacterium zeae* strain J20M2RI	*Microbacterium zeae*	99%	99.88%	MT409535.1
10		*Brevundimonas* sp. strain SH-3.2-R-10	*Brevundimonas* sp.	100%	99.70%	MN784238.1
11		*Brevundimonas* sp. strain SH-3.2-R-10	*Brevundimonas* sp.	100%	100.00%	MN784238.1
12		*Bacillus safensis* strain SRCM125915	*Bacillus safensis*	100%	100.00%	CP116774.1
17		*Microbacterium testaceum* strain DK33	*Microbacterium testaceum*	100%	99.92%	MT534540.1
18		*Sphingomonas melonis* strain IARI-CW-25	*Sphingomonas melonis*	100%	99.75%	JF343163.1
19		*Microbacterium testaceum* strain DK33	*Microbacterium testaceum*	100%	99.91%	MT534540.1
20		*Microbacterium testaceum* strain DK33	*Microbacterium testaceum*	100%	99.92%	MT534540.1
21		*Microbacterium proteolyticum* strain OsEnb_PLM_L82	*Microbacterium proteolyticum*	100%	99.92%	MN889295.1

24		*Sphingomonas melonis* strain IARI-CW-25	*Sphingomonas melonis*	100%	99.84%	JF343163.1
66		*Bacillus safensis* strain SRCM125915	*Bacillus safensis*	100%	100.00%	CP116774.1
78		*Microbacterium* sp. strain FW305-3-2-15-F-LB2	*Microbacterium* sp.	100%	99.92%	MH795568.1
59	Mo	*Bacillus acidiceler* strain N15121	*Bacillus acidicelers*	100%	100.00%	MK389284.1
60		*Bacillus acidiceler* strain N15121	*Bacillus acidicelers*	100%	100.00%	MK389284.1
65		*Bacillus tropicus* strain FDAARGOS_782	*Bacillus tropicus*	100%	99.92%	CP053955.1
79		*Bacillus acidiceler* strain CBD119	*Bacillus acidicelers*	100%	100.00%	MN543770.1
14	NC	*Rhodococcus yunnanensis* strain OsEnb_PLM_L24	*Rhodococcus yunnanensis*	100%	99.92%	MN889263.1
25		*Microbacterium testaceum* strain DK33	*Microbacterium testaceum*	100%	99.92%	MT534540.1
26		*Rhodococcus yunnanensis* strain OsEnb_PLM_L24	*Rhodococcus yunnanensis*	100%	100.00%	MN889263.1
27		*Rhodococcus* sp. strain BR(2)3	*Rhodococcus* sp.	100%	99.84%	OL693037.1
28		*Rhodococcus yunnanensis* strain OsEnb_PLM_L24	*Rhodococcus yunnanensis*	100%	100.00%	MN889263.1

29		*Microbacterium testaceum* strain BY-1	*Microbacterium testaceum*	100%	99.92%	MT605456.1
38		*Microbacterium testaceum* strain DK33	*Microbacterium testaceum*	100%	100.00%	MT534540.1
63		*Bacillus* sp. (in: Bacteria) strain CS14	*Bacillus* sp. (in: Bacteria)	100%	100.00%	MT584789.1
68		*Bacillus altitudinis* strain P5.15	*Bacillus altitudinis*	100%	100.00%	OQ295976.1
76		*Bacillus* sp. EAAC32	*Bacillus* sp. EAAC32	100%	99.92%	KT151920.1
77		*Microbacterium testaceum* strain BY-1	*Microbacterium testaceum*	100%	100.00%	MT605456.1
52	NE	*Microbacterium testaceum* strain DK33	*Microbacterium testaceum*	100%	100.00%	MT534540.1
54		*Bacillus safensis* strain SRCM125915	*Bacillus safensis*	100%	100.00%	CP116774.1
55		*Microbacterium testaceum* strain BY-1	*Microbacterium testaceum*	100%	100.00%	MT605456.1
67		*Bacillus cereus* strain PSR21	*Bacillus cereus*	100%	100.00%	ON860698.1
74		*Paenibacillus* sp. 9HE-11	*Paenibacillus* sp.	100%	99.59%	LC338050.1
80		*Kocuria* sp. T35-5	*Kocuria* sp.	100%	100.00%	LC556325.1
